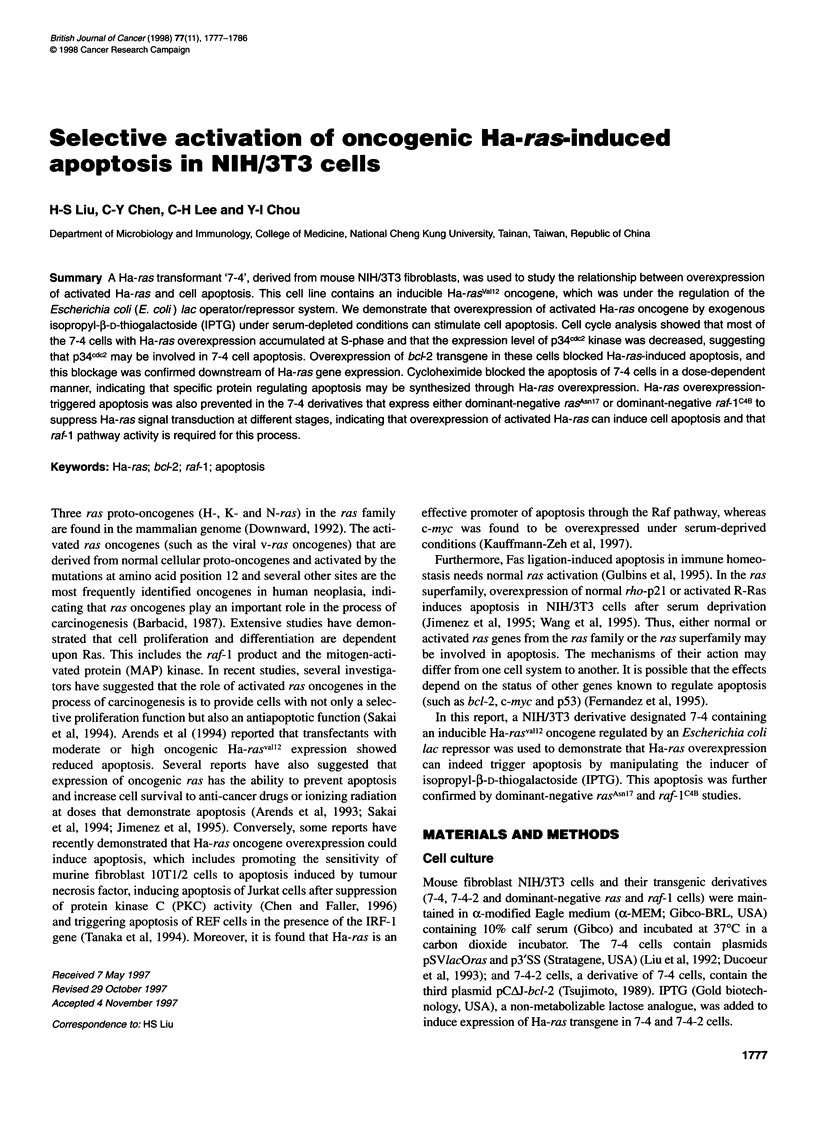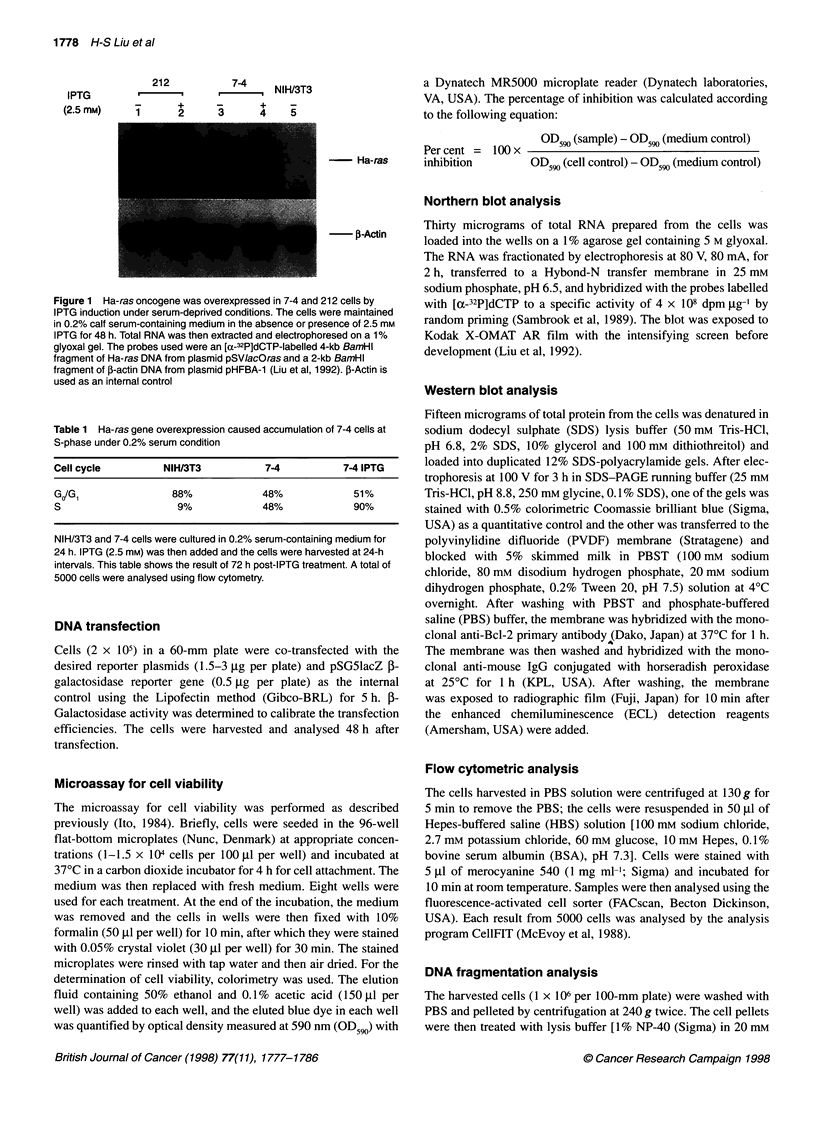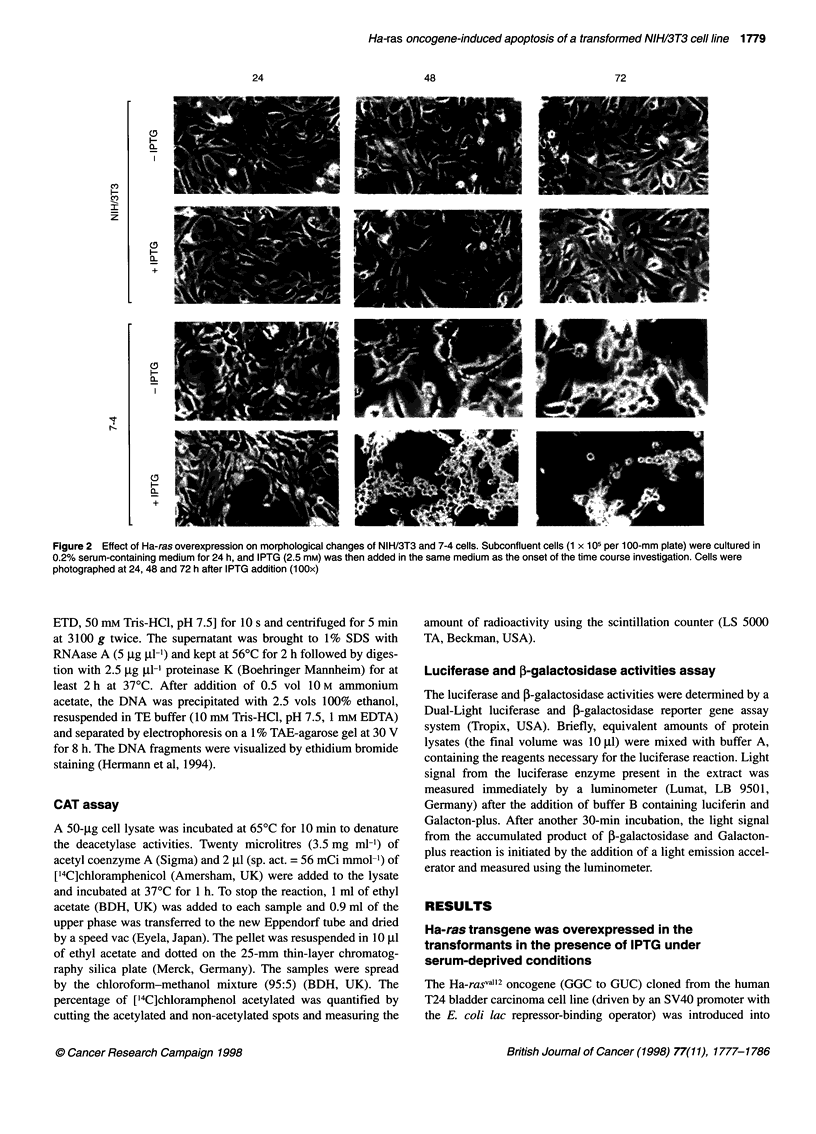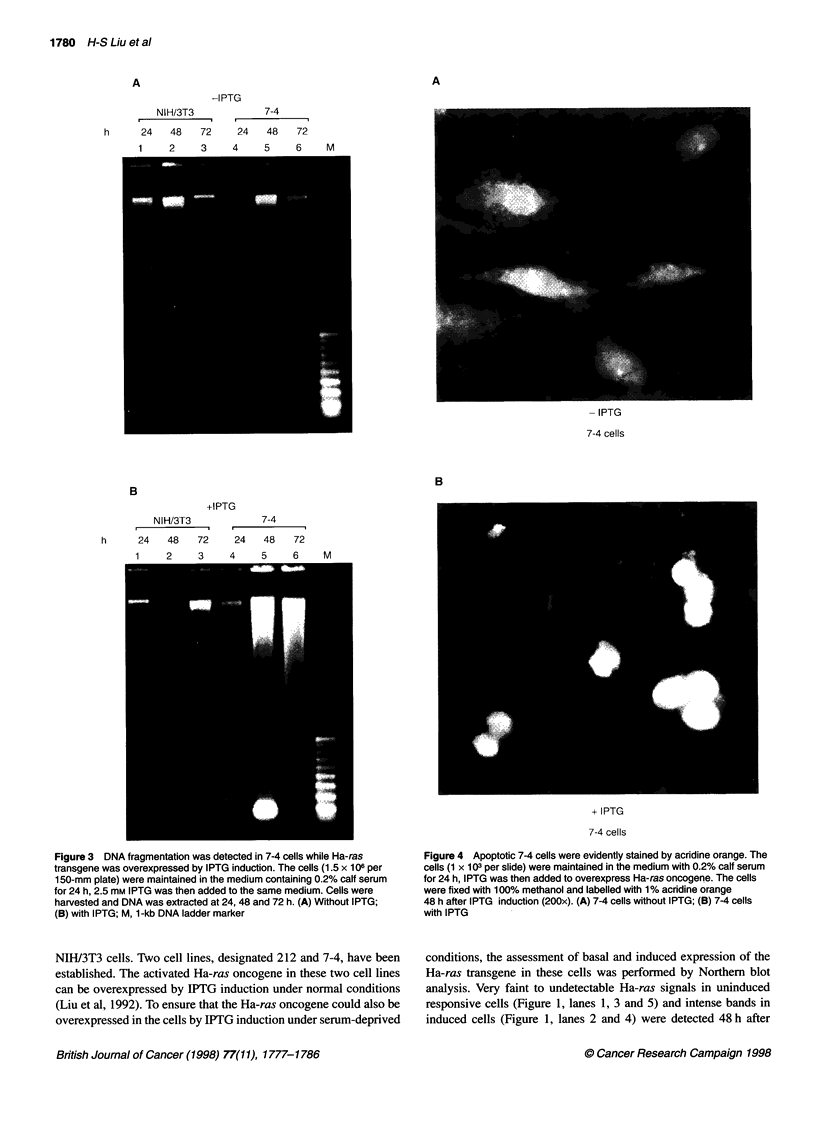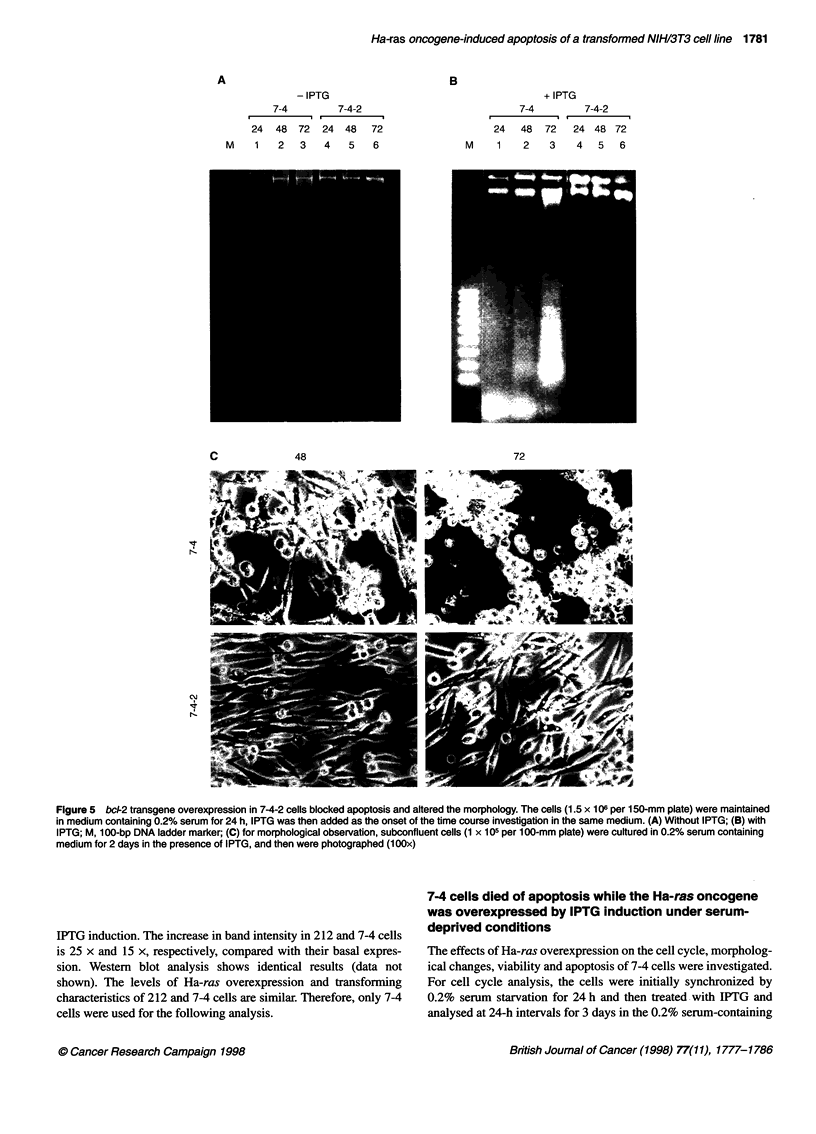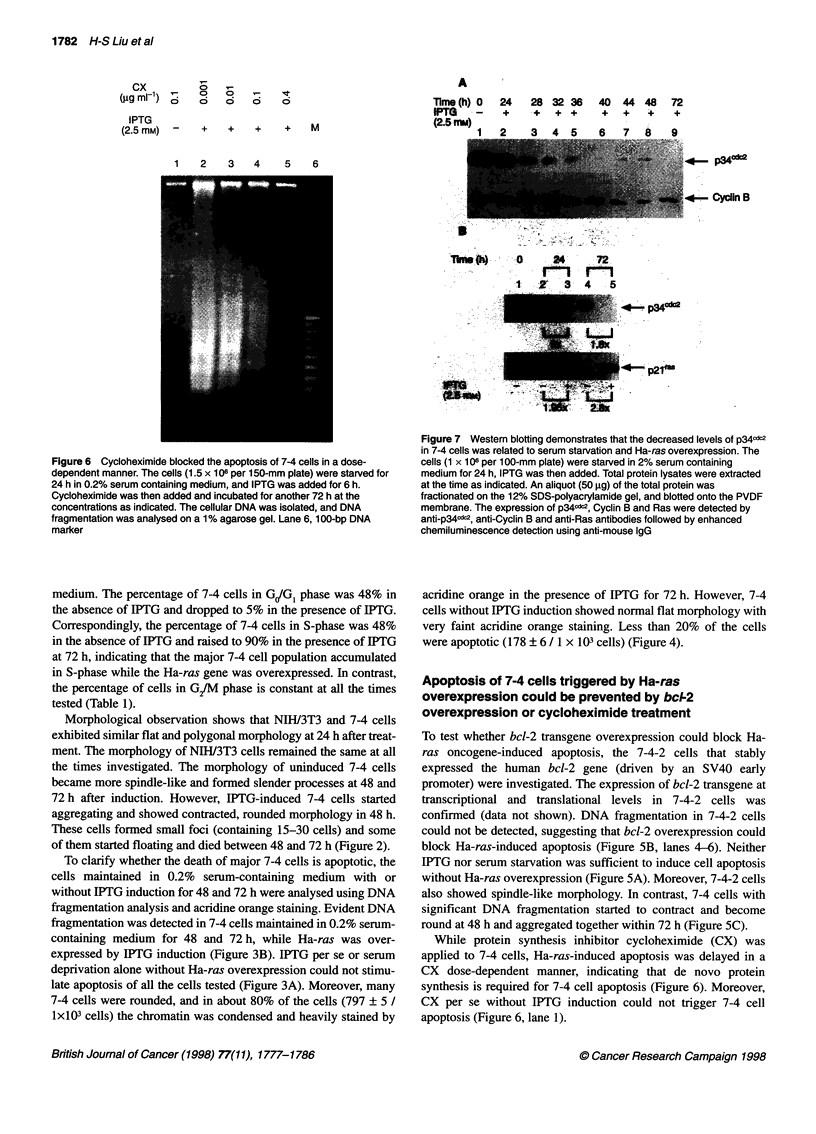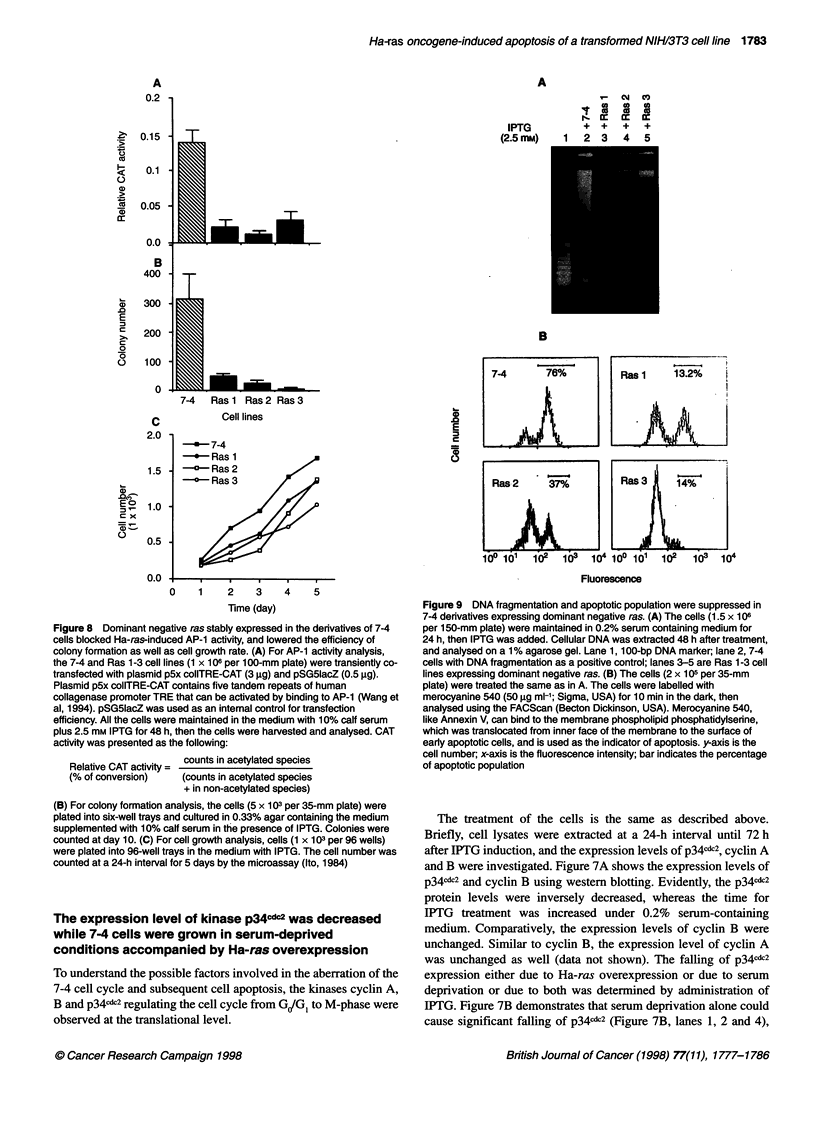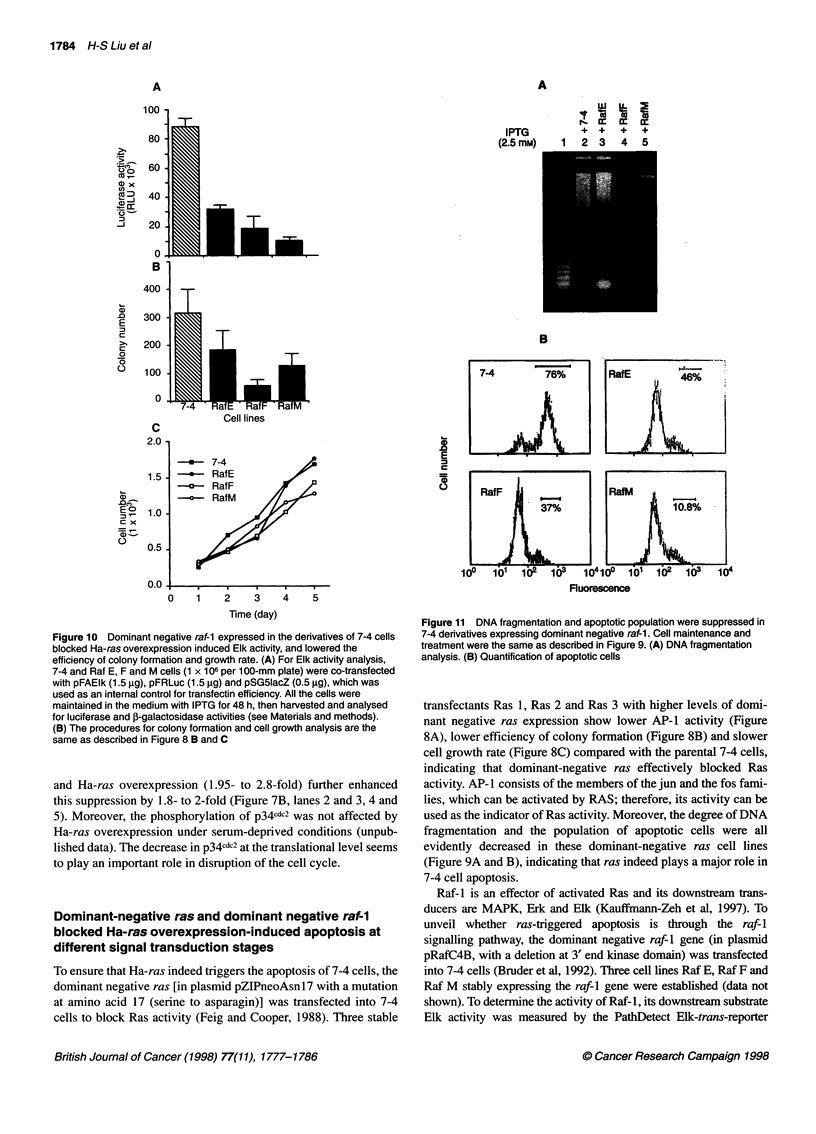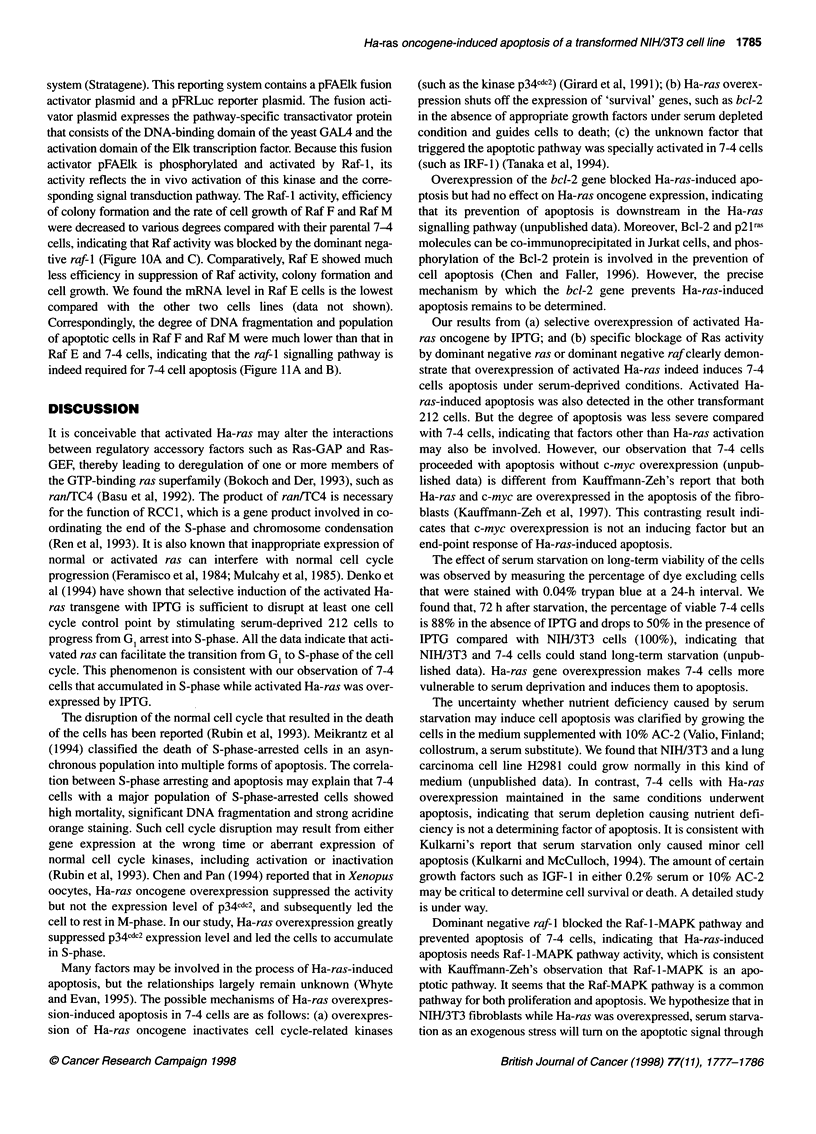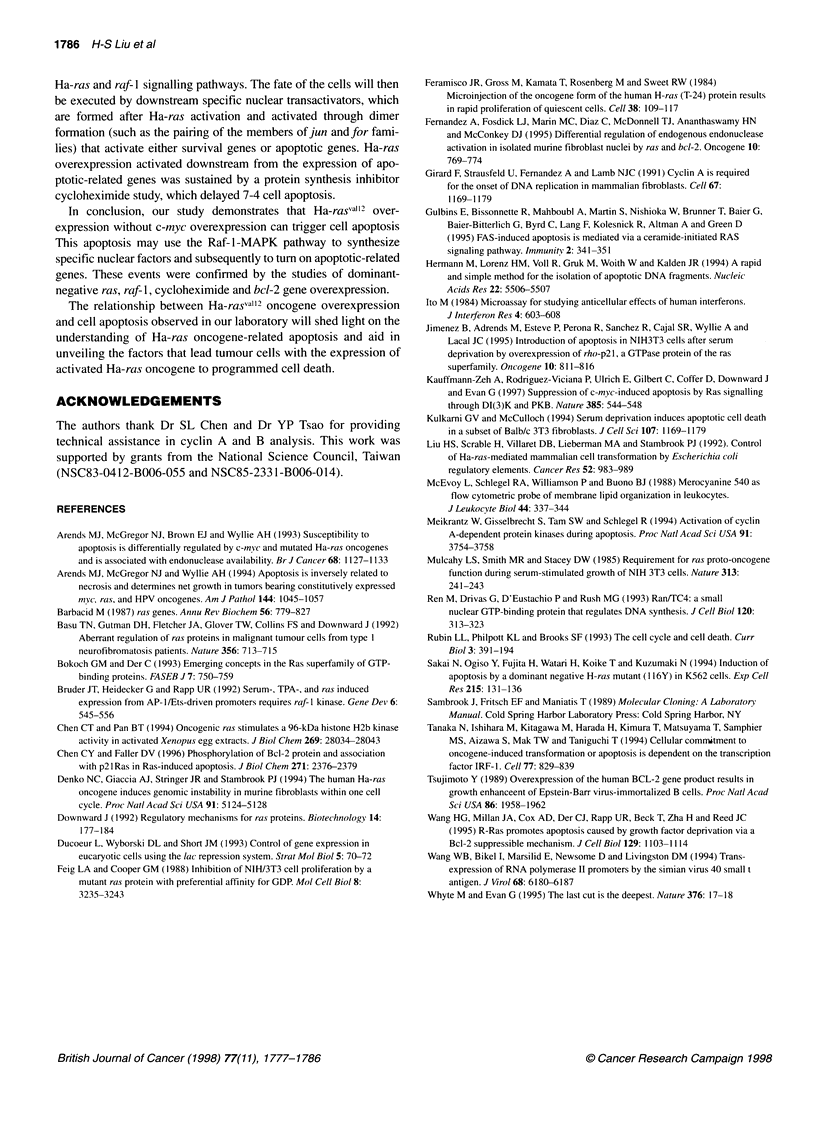# Selective activation of oncogenic Ha-ras-induced apoptosis in NIH/3T3 cells.

**DOI:** 10.1038/bjc.1998.296

**Published:** 1998-06

**Authors:** H. S. Liu, C. Y. Chen, C. H. Lee, Y. I. Chou

**Affiliations:** Department of Microbiology and Immunology, College of Medicine, National Cheng Kung University, Tainan, Taiwan, Republic of China.

## Abstract

**Images:**


*British Journal of Cancer* (1998) **77**(11), 1777–1786
© 1998 Cancer Research Campaign

### Selective activation of oncogenic Ha-*ras*-induced apoptosis in NIH/3T3 cells

**H-S Liu, C-Y Chen, C-H Lee and Y-I Chou**

Department of Microbiology and Immunology, College of Medicine, National Cheng Kung University, Tainan, Taiwan, Republic of China

**Summary** A Ha-*ras* transformant '7-4', derived from mouse NIH/3T3 fibroblasts, was used to study the relationship between overexpression of activated Ha-*ras* and cell apoptosis. This cell line contains an inducible Ha-*ras*$^{Val12}$ oncogene, which was under the regulation of the *Escherichia coli* (*E. coli*) *lac* operator/repressor system. We demonstrate that overexpression of activated Ha-*ras* oncogene by exogenous isopropyl-β-D-thiogalactoside (IPTG) under serum-depleted conditions can stimulate cell apoptosis. Cell cycle analysis showed that most of the 7-4 cells with Ha-*ras* overexpression accumulated at S-phase and that the expression level of p34$^{cdc2}$ kinase was decreased, suggesting that p34$^{cdc2}$ may be involved in 7-4 cell apoptosis. Overexpression of *bcl*-2 transgene in these cells blocked Ha-*ras*-induced apoptosis, and this blockage was confirmed downstream of Ha-*ras* gene expression. Cycloheximide blocked the apoptosis of 7-4 cells in a dose-dependent manner, indicating that specific protein regulating apoptosis may be synthesized through Ha-*ras* overexpression. Ha-*ras* overexpression-triggered apoptosis was also prevented in the 7-4 derivatives that express either dominant-negative *ras*$^{Asn17}$ or dominant-negative *raf*-1$^{C4B}$ to suppress Ha-*ras* signal transduction at different stages, indicating that overexpression of activated Ha-*ras* can induce cell apoptosis and that *raf*-1 pathway activity is required for this process.

**Keywords:** Ha-*ras*; *bcl*-2; *raf*-1; apoptosis

Three *ras* proto-oncogenes (H-, K- and N-*ras*) in the *ras* family are found in the mammalian genome (Downward, 1992). The activated *ras* oncogenes (such as the viral v-*ras* oncogenes) that are derived from normal cellular proto-oncogenes and activated by the mutations at amino acid position 12 and several other sites are the most frequently identified oncogenes in human neoplasia, indicating that *ras* oncogenes play an important role in the process of carcinogenesis (Barbacid, 1987). Extensive studies have demonstrated that cell proliferation and differentiation are dependent upon Ras. This includes the *raf*-1 product and the mitogen-activated protein (MAP) kinase. In recent studies, several investigators have suggested that the role of activated *ras* oncogenes in the process of carcinogenesis is to provide cells with not only a selective proliferation function but also an antiapoptotic function (Sakai et al, 1994). Arends et al (1994) reported that transfectants with moderate or high oncogenic Ha-*ras*$^{val12}$ expression showed reduced apoptosis. Several reports have also suggested that expression of oncogenic *ras* has the ability to prevent apoptosis and increase cell survival to anti-cancer drugs or ionizing radiation at doses that demonstrate apoptosis (Arends et al, 1993; Sakai et al, 1994; Jimenez et al, 1995). Conversely, some reports have recently demonstrated that Ha-*ras* oncogene overexpression could induce apoptosis, which includes promoting the sensitivity of murine fibroblast 10T1/2 cells to apoptosis induced by tumour necrosis factor, inducing apoptosis of Jurkat cells after suppression of protein kinase C (PKC) activity (Chen and Faller, 1996) and triggering apoptosis of REF cells in the presence of the IRF-1 gene (Tanaka et al, 1994). Moreover, it is found that Ha-*ras* is an effective promoter of apoptosis through the Raf pathway, whereas c-*myc* was found to be overexpressed under serum-deprived conditions (Kauffmann-Zeh et al, 1997).

Furthermore, Fas ligation-induced apoptosis in immune homeostasis needs normal *ras* activation (Gulbins et al, 1995). In the *ras* superfamily, overexpression of normal *rho*-p21 or activated R-Ras induces apoptosis in NIH/3T3 cells after serum deprivation (Jimenez et al, 1995; Wang et al, 1995). Thus, either normal or activated *ras* genes from the *ras* family or the *ras* superfamily may be involved in apoptosis. The mechanisms of their action may differ from one cell system to another. It is possible that the effects depend on the status of other genes known to regulate apoptosis (such as *bcl*-2, c-*myc* and p53) (Fernandez et al, 1995).

In this report, a NIH/3T3 derivative designated 7-4 containing an inducible Ha-*ras*$^{val12}$ oncogene regulated by an *Escherichia coli lac* repressor was used to demonstrate that Ha-*ras* overexpression can indeed trigger apoptosis by manipulating the inducer of isopropyl-β-D-thiogalactoside (IPTG). This apoptosis was further confirmed by dominant-negative *ras*$^{Asn17}$ and *raf*-1$^{C4B}$ studies.

### MATERIALS AND METHODS

### Cell culture

Mouse fibroblast NIH/3T3 cells and their transgenic derivatives (7-4, 7-4-2 and dominant-negative *ras* and *raf*-1 cells) were maintained in α-modified Eagle medium (α-MEM; Gibco-BRL, USA) containing 10% calf serum (Gibco) and incubated at 37°C in a carbon dioxide incubator. The 7-4 cells contain plasmids pSV*lacO**ras* and p3′SS (Stratagene, USA) (Liu et al, 1992; Ducoeur et al, 1993); and 7-4-2 cells, a derivative of 7-4 cells, contain the third plasmid pCΔJ-*bcl*-2 (Tsujimoto, 1989). IPTG (Gold biotechnology, USA), a non-metabolizable lactose analogue, was added to induce expression of Ha-*ras* transgene in 7-4 and 7-4-2 cells.

*Received 7 May 1997*
*Revised 29 October 1997*
*Accepted 4 November 1997*

*Correspondence to:* HS Liu

**Figure 1** Ha-*ras* oncogene was overexpressed in 7-4 and 212 cells by IPTG induction under serum-deprived conditions. The cells were maintained in 0.2% calf serum-containing medium in the absence or presence of 2.5 mM IPTG for 48 h. Total RNA was then extracted and electrophoresed on a 1% glyoxal gel. The probes used were an [α-$^{32}$P]dCTP-labelled 4-kb *Bam*HI fragment of Ha-*ras* DNA from plasmid pSV*lac*O*ras* and a 2-kb *Bam*HI fragment of β-actin DNA from plasmid pHFBA-1 (Liu et al, 1992). β-Actin is used as an internal control

**Table 1** Ha-*ras* gene overexpression caused accumulation of 7-4 cells at S-phase under 0.2% serum condition

| Cell cycle | NIH/3T3 | 7-4 | 7-4 IPTG |
|---|---|---|---|
| $G_0/G_1$ | 88% | 48% | 51% |
| S | 9% | 48% | 90% |

NIH/3T3 and 7-4 cells were cultured in 0.2% serum-containing medium for 24 h. IPTG (2.5 mM) was then added and the cells were harvested at 24-h intervals. This table shows the result of 72 h post-IPTG treatment. A total of 5000 cells were analysed using flow cytometry.

### DNA transfection

Cells (2 × 10$^5$) in a 60-mm plate were co-transfected with the desired reporter plasmids (1.5–3 μg per plate) and pSG5lacZ β-galactosidase reporter gene (0.5 μg per plate) as the internal control using the Lipofectin method (Gibco-BRL) for 5 h. β-Galactosidase activity was determined to calibrate the transfection efficiencies. The cells were harvested and analysed 48 h after transfection.

### Microassay for cell viability

The microassay for cell viability was performed as described previously (Ito, 1984). Briefly, cells were seeded in the 96-well flat-bottom microplates (Nunc, Denmark) at appropriate concentrations (1–1.5 × 10$^4$ cells per 100 μl per well) and incubated at 37°C in a carbon dioxide incubator for 4 h for cell attachment. The medium was then replaced with fresh medium. Eight wells were used for each treatment. At the end of the incubation, the medium was removed and the cells in wells were then fixed with 10% formalin (50 μl per well) for 10 min, after which they were stained with 0.05% crystal violet (30 μl per well) for 30 min. The stained microplates were rinsed with tap water and then air dried. For the determination of cell viability, colorimetry was used. The elution fluid containing 50% ethanol and 0.1% acetic acid (150 μl per well) was added to each well, and the eluted blue dye in each well was quantified by optical density measured at 590 nm ($OD_{590}$) with a Dynatech MR5000 microplate reader (Dynatech laboratories, VA, USA). The percentage of inhibition was calculated according to the following equation:

$$\text{Per cent inhibition} = 100 \times \frac{OD_{590}\,(\text{sample}) - OD_{590}\,(\text{medium control})}{OD_{590}\,(\text{cell control}) - OD_{590}\,(\text{medium control})}$$

### Northern blot analysis

Thirty micrograms of total RNA prepared from the cells was loaded into the wells on a 1% agarose gel containing 5 M glyoxal. The RNA was fractionated by electrophoresis at 80 V, 80 mA, for 2 h, transferred to a Hybond-N transfer membrane in 25 mM sodium phosphate, pH 6.5, and hybridized with the probes labelled with [α-$^{32}$P]dCTP to a specific activity of 4 × 10$^8$ dpm μg$^{-1}$ by random priming (Sambrook et al, 1989). The blot was exposed to Kodak X-OMAT AR film with the intensifying screen before development (Liu et al, 1992).

### Western blot analysis

Fifteen micrograms of total protein from the cells was denatured in sodium dodecyl sulphate (SDS) lysis buffer (50 mM Tris-HCl, pH 6.8, 2% SDS, 10% glycerol and 100 mM dithiothreitol) and loaded into duplicated 12% SDS-polyacrylamide gels. After electrophoresis at 100 V for 3 h in SDS–PAGE running buffer (25 mM Tris-HCl, pH 8.8, 250 mM glycine, 0.1% SDS), one of the gels was stained with 0.5% colorimetric Coomassie brilliant blue (Sigma, USA) as a quantitative control and the other was transferred to the polyvinylidine difluoride (PVDF) membrane (Stratagene) and blocked with 5% skimmed milk in PBST (100 mM sodium chloride, 80 mM disodium hydrogen phosphate, 20 mM sodium dihydrogen phosphate, 0.2% Tween 20, pH 7.5) solution at 4°C overnight. After washing with PBST and phosphate-buffered saline (PBS) buffer, the membrane was hybridized with the monoclonal anti-Bcl-2 primary antibody (Dako, Japan) at 37°C for 1 h. The membrane was then washed and hybridized with the monoclonal anti-mouse IgG conjugated with horseradish peroxidase at 25°C for 1 h (KPL, USA). After washing, the membrane was exposed to radiographic film (Fuji, Japan) for 10 min after the enhanced chemiluminescence (ECL) detection reagents (Amersham, USA) were added.

### Flow cytometric analysis

The cells harvested in PBS solution were centrifuged at 130 ***g*** for 5 min to remove the PBS; the cells were resuspended in 50 μl of Hepes-buffered saline (HBS) solution [100 mM sodium chloride, 2.7 mM potassium chloride, 60 mM glucose, 10 mM Hepes, 0.1% bovine serum albumin (BSA), pH 7.3]. Cells were stained with 5 μl of merocyanine 540 (1 mg ml$^{-1}$; Sigma) and incubated for 10 min at room temperature. Samples were then analysed using the fluorescence-activated cell sorter (FACscan, Becton Dickinson, USA). Each result from 5000 cells was analysed by the analysis program CellFIT (McEvoy et al, 1988).

### DNA fragmentation analysis

The harvested cells (1 × 10$^6$ per 100-mm plate) were washed with PBS and pelleted by centrifugation at 240 ***g*** twice. The cell pellets were then treated with lysis buffer [1% NP-40 (Sigma) in 20 mM

© Cancer Research Campaign 1998

**Figure 2** Effect of Ha-*ras* overexpression on morphological changes of NIH/3T3 and 7-4 cells. Subconfluent cells ($1 \times 10^5$ per 100-mm plate) were cultured in 0.2% serum-containing medium for 24 h, and IPTG (2.5 mM) was then added in the same medium as the onset of the time course investigation. Cells were photographed at 24, 48 and 72 h after IPTG addition (100×)

ETD, 50 mM Tris-HCl, pH 7.5] for 10 s and centrifuged for 5 min at 3100 ***g*** twice. The supernatant was brought to 1% SDS with RNAase A (5 μg $\mu l^{-1}$) and kept at 56°C for 2 h followed by digestion with 2.5 μg $\mu l^{-1}$ proteinase K (Boehringer Mannheim) for at least 2 h at 37°C. After addition of 0.5 vol 10 M ammonium acetate, the DNA was precipitated with 2.5 vols 100% ethanol, resuspended in TE buffer (10 mM Tris-HCl, pH 7.5, 1 mM EDTA) and separated by electrophoresis on a 1% TAE-agarose gel at 30 V for 8 h. The DNA fragments were visualized by ethidium bromide staining (Hermann et al, 1994).

### CAT assay

A 50-μg cell lysate was incubated at 65°C for 10 min to denature the deacetylase activities. Twenty microlitres (3.5 mg $ml^{-1}$) of acetyl coenzyme A (Sigma) and 2 μl (sp. act. = 56 mCi $mmol^{-1}$) of [$^{14}C$]chloramphenicol (Amersham, UK) were added to the lysate and incubated at 37°C for 1 h. To stop the reaction, 1 ml of ethyl acetate (BDH, UK) was added to each sample and 0.9 ml of the upper phase was transferred to the new Eppendorf tube and dried by a speed vac (Eyela, Japan). The pellet was resuspended in 10 μl of ethyl acetate and dotted on the 25-mm thin-layer chromatography silica plate (Merck, Germany). The samples were spread by the chloroform–methanol mixture (95:5) (BDH, UK). The percentage of [$^{14}C$]chloramphenol acetylated was quantified by cutting the acetylated and non-acetylated spots and measuring the amount of radioactivity using the scintillation counter (LS 5000 TA, Beckman, USA).

### Luciferase and β-galactosidase activities assay

The luciferase and β-galactosidase activities were determined by a Dual-Light luciferase and β-galactosidase reporter gene assay system (Tropix, USA). Briefly, equivalent amounts of protein lysates (the final volume was 10 μl) were mixed with buffer A, containing the reagents necessary for the luciferase reaction. Light signal from the luciferase enzyme present in the extract was measured immediately by a luminometer (Lumat, LB 9501, Germany) after the addition of buffer B containing luciferin and Galacton-plus. After another 30-min incubation, the light signal from the accumulated product of β-galactosidase and Galacton-plus reaction is initiated by the addition of a light emission accelerator and measured using the luminometer.

### RESULTS

### Ha-*ras* transgene was overexpressed in the transformants in the presence of IPTG under serum-deprived conditions

The Ha-*ras*$^{val12}$ oncogene (GGC to GUC) cloned from the human T24 bladder carcinoma cell line (driven by an SV40 promoter with the *E. coli lac* repressor-binding operator) was introduced into

**Figure 3** DNA fragmentation was detected in 7-4 cells while Ha-*ras* transgene was overexpressed by IPTG induction. The cells ($1.5 \times 10^6$ per 150-mm plate) were maintained in the medium containing 0.2% calf serum for 24 h, 2.5 mM IPTG was then added to the same medium. Cells were harvested and DNA was extracted at 24, 48 and 72 h. (**A**) Without IPTG; (**B**) with IPTG; M, 1-kb DNA ladder marker

**Figure 4** Apoptotic 7-4 cells were evidently stained by acridine orange. The cells ($1 \times 10^3$ per slide) were maintained in the medium with 0.2% calf serum for 24 h, IPTG was then added to overexpress Ha-*ras* oncogene. The cells were fixed with 100% methanol and labelled with 1% acridine orange 48 h after IPTG induction (200×). (**A**) 7-4 cells without IPTG; (**B**) 7-4 cells with IPTG

NIH/3T3 cells. Two cell lines, designated 212 and 7-4, have been established. The activated Ha-*ras* oncogene in these two cell lines can be overexpressed by IPTG induction under normal conditions (Liu et al, 1992). To ensure that the Ha-*ras* oncogene could also be overexpressed in the cells by IPTG induction under serum-deprived conditions, the assessment of basal and induced expression of the Ha-*ras* transgene in these cells was performed by Northern blot analysis. Very faint to undetectable Ha-*ras* signals in uninduced responsive cells (Figure 1, lanes 1, 3 and 5) and intense bands in induced cells (Figure 1, lanes 2 and 4) were detected 48 h after

 © Cancer Research Campaign 1998

**Figure 5** *bcl*-2 transgene overexpression in 7-4-2 cells blocked apoptosis and altered the morphology. The cells ($1.5 \times 10^6$ per 150-mm plate) were maintained in medium containing 0.2% serum for 24 h, IPTG was then added as the onset of the time course investigation in the same medium. (**A**) Without IPTG; (**B**) with IPTG; M, 100-bp DNA ladder marker; (**C**) for morphological observation, subconfluent cells ($1 \times 10^5$ per 100-mm plate) were cultured in 0.2% serum containing medium for 2 days in the presence of IPTG, and then were photographed (100×)

IPTG induction. The increase in band intensity in 212 and 7-4 cells is 25 × and 15 ×, respectively, compared with their basal expression. Western blot analysis shows identical results (data not shown). The levels of Ha-*ras* overexpression and transforming characteristics of 212 and 7-4 cells are similar. Therefore, only 7-4 cells were used for the following analysis.

### 7-4 cells died of apoptosis while the Ha-*ras* oncogene was overexpressed by IPTG induction under serum-deprived conditions

The effects of Ha-*ras* overexpression on the cell cycle, morphological changes, viability and apoptosis of 7-4 cells were investigated. For cell cycle analysis, the cells were initially synchronized by 0.2% serum starvation for 24 h and then treated with IPTG and analysed at 24-h intervals for 3 days in the 0.2% serum-containing

© Cancer Research Campaign 1998

**Figure 6** Cycloheximide blocked the apoptosis of 7-4 cells in a dose-dependent manner. The cells (1.5 × $10^6$ per 150-mm plate) were starved for 24 h in 0.2% serum containing medium, and IPTG was added for 6 h. Cycloheximide was then added and incubated for another 72 h at the concentrations as indicated. The cellular DNA was isolated, and DNA fragmentation was analysed on a 1% agarose gel. Lane 6, 100-bp DNA marker

**Figure 7** Western blotting demonstrates that the decreased levels of $p34^{cdc2}$ in 7-4 cells was related to serum starvation and Ha-*ras* overexpression. The cells (1 × $10^6$ per 100-mm plate) were starved in 2% serum containing medium for 24 h, IPTG was then added. Total protein lysates were extracted at the time as indicated. An aliquot (50 μg) of the total protein was fractionated on the 12% SDS-polyacrylamide gel, and blotted onto the PVDF membrane. The expression of $p34^{cdc2}$, Cyclin B and Ras were detected by anti-$p34^{cdc2}$, anti-Cyclin B and anti-Ras antibodies followed by enhanced chemiluminescence detection using anti-mouse IgG

medium. The percentage of 7-4 cells in $G_0/G_1$ phase was 48% in the absence of IPTG and dropped to 5% in the presence of IPTG. Correspondingly, the percentage of 7-4 cells in S-phase was 48% in the absence of IPTG and raised to 90% in the presence of IPTG at 72 h, indicating that the major 7-4 cell population accumulated in S-phase while the Ha-*ras* gene was overexpressed. In contrast, the percentage of cells in $G_2/M$ phase is constant at all the times tested (Table 1).

Morphological observation shows that NIH/3T3 and 7-4 cells exhibited similar flat and polygonal morphology at 24 h after treatment. The morphology of NIH/3T3 cells remained the same at all the times investigated. The morphology of uninduced 7-4 cells became more spindle-like and formed slender processes at 48 and 72 h after induction. However, IPTG-induced 7-4 cells started aggregating and showed contracted, rounded morphology in 48 h. These cells formed small foci (containing 15–30 cells) and some of them started floating and died between 48 and 72 h (Figure 2).

To clarify whether the death of major 7-4 cells is apoptotic, the cells maintained in 0.2% serum-containing medium with or without IPTG induction for 48 and 72 h were analysed using DNA fragmentation analysis and acridine orange staining. Evident DNA fragmentation was detected in 7-4 cells maintained in 0.2% serum-containing medium for 48 and 72 h, while Ha-*ras* was overexpressed by IPTG induction (Figure 3B). IPTG per se or serum deprivation alone without Ha-*ras* overexpression could not stimulate apoptosis of all the cells tested (Figure 3A). Moreover, many 7-4 cells were rounded, and in about 80% of the cells (797 ± 5 / 1×$10^3$ cells) the chromatin was condensed and heavily stained by acridine orange in the presence of IPTG for 72 h. However, 7-4 cells without IPTG induction showed normal flat morphology with very faint acridine orange staining. Less than 20% of the cells were apoptotic (178 ± 6 / 1 × $10^3$ cells) (Figure 4).

### Apoptosis of 7-4 cells triggered by Ha-*ras* overexpression could be prevented by *bcl*-2 overexpression or cycloheximide treatment

To test whether *bcl*-2 transgene overexpression could block Ha-*ras* oncogene-induced apoptosis, the 7-4-2 cells that stably expressed the human *bcl*-2 gene (driven by an SV40 early promoter) were investigated. The expression of *bcl*-2 transgene at transcriptional and translational levels in 7-4-2 cells was confirmed (data not shown). DNA fragmentation in 7-4-2 cells could not be detected, suggesting that *bcl*-2 overexpression could block Ha-*ras*-induced apoptosis (Figure 5B, lanes 4–6). Neither IPTG nor serum starvation was sufficient to induce cell apoptosis without Ha-*ras* overexpression (Figure 5A). Moreover, 7-4-2 cells also showed spindle-like morphology. In contrast, 7-4 cells with significant DNA fragmentation started to contract and become round at 48 h and aggregated together within 72 h (Figure 5C).

While protein synthesis inhibitor cycloheximide (CX) was applied to 7-4 cells, Ha-*ras*-induced apoptosis was delayed in a CX dose-dependent manner, indicating that de novo protein synthesis is required for 7-4 cell apoptosis (Figure 6). Moreover, CX per se without IPTG induction could not trigger 7-4 cell apoptosis (Figure 6, lane 1).

 © Cancer Research Campaign 1998

**Figure 8** Dominant negative *ras* stably expressed in the derivatives of 7-4 cells blocked Ha-*ras*-induced AP-1 activity, and lowered the efficiency of colony formation as well as cell growth rate. (**A**) For AP-1 activity analysis, the 7-4 and Ras 1-3 cell lines (1 × 10$^6$ per 100-mm plate) were transiently co-transfected with plasmid p5x collTRE-CAT (3 μg) and pSG5lacZ (0.5 μg). Plasmid p5x collTRE-CAT contains five tandem repeats of human collagenase promoter TRE that can be activated by binding to AP-1 (Wang et al, 1994). pSG5lacZ was used as an internal control for transfection efficiency. All the cells were maintained in the medium with 10% calf serum plus 2.5 mM IPTG for 48 h, then the cells were harvested and analysed. CAT activity was presented as the following:

$$\text{Relative CAT activity (\% of conversion)} = \frac{\text{counts in acetylated species}}{\text{(counts in acetylated species + in non-acetylated species)}}$$

(**B**) For colony formation analysis, the cells (5 × 10$^3$ per 35-mm plate) were plated into six-well trays and cultured in 0.33% agar containing the medium supplemented with 10% calf serum in the presence of IPTG. Colonies were counted at day 10. (**C**) For cell growth analysis, cells (1 × 10$^3$ per 96 wells) were plated into 96-well trays in the medium with IPTG. The cell number was counted at a 24-h interval for 5 days by the microassay (Ito, 1984)

**Figure 9** DNA fragmentation and apoptotic population were suppressed in 7-4 derivatives expressing dominant negative *ras*. (**A**) The cells (1.5 × 10$^6$ per 150-mm plate) were maintained in 0.2% serum containing medium for 24 h, then IPTG was added. Cellular DNA was extracted 48 h after treatment, and analysed on a 1% agarose gel. Lane 1, 100-bp DNA marker; lane 2, 7-4 cells with DNA fragmentation as a positive control; lanes 3–5 are Ras 1-3 cell lines expressing dominant negative *ras*. (**B**) The cells (2 × 10$^5$ per 35-mm plate) were treated the same as in A. The cells were labelled with merocyanine 540 (50 μg ml$^{-1}$; Sigma, USA) for 10 min in the dark, then analysed using the FACScan (Becton Dickinson, USA). Merocyanine 540, like Annexin V, can bind to the membrane phospholipid phosphatidylserine, which was translocated from inner face of the membrane to the surface of early apoptotic cells, and is used as the indicator of apoptosis. *y*-axis is the cell number; *x*-axis is the fluorescence intensity; bar indicates the percentage of apoptotic population

### The expression level of kinase p34$^{cdc2}$ was decreased while 7-4 cells were grown in serum-deprived conditions accompanied by Ha-*ras* overexpression

To understand the possible factors involved in the aberration of the 7-4 cell cycle and subsequent cell apoptosis, the kinases cyclin A, B and p34$^{cdc2}$ regulating the cell cycle from $G_0/G_1$ to M-phase were observed at the translational level.

The treatment of the cells is the same as described above. Briefly, cell lysates were extracted at a 24-h interval until 72 h after IPTG induction, and the expression levels of p34$^{cdc2}$, cyclin A and B were investigated. Figure 7A shows the expression levels of p34$^{cdc2}$ and cyclin B using western blotting. Evidently, the p34$^{cdc2}$ protein levels were inversely decreased, whereas the time for IPTG treatment was increased under 0.2% serum-containing medium. Comparatively, the expression levels of cyclin B were unchanged. Similar to cyclin B, the expression level of cyclin A was unchanged as well (data not shown). The falling of p34$^{cdc2}$ expression either due to Ha-*ras* overexpression or due to serum deprivation or due to both was determined by administration of IPTG. Figure 7B demonstrates that serum deprivation alone could cause significant falling of p34$^{cdc2}$ (Figure 7B, lanes 1, 2 and 4),

© Cancer Research Campaign 1998

**Figure 10** Dominant negative *raf*-1 expressed in the derivatives of 7-4 cells blocked Ha-*ras* overexpression induced Elk activity, and lowered the efficiency of colony formation and growth rate. (**A**) For Elk activity analysis, 7-4 and Raf E, F and M cells (1 x $10^6$ per 100-mm plate) were co-transfected with pFAElk (1.5 μg), pFRLuc (1.5 μg) and pSG5lacZ (0.5 μg), which was used as an internal control for transfectin efficiency. All the cells were maintained in the medium with IPTG for 48 h, then harvested and analysed for luciferase and β-galactosidase activities (see Materials and methods). (**B**) The procedures for colony formation and cell growth analysis are the same as described in Figure 8 **B** and **C**

**Figure 11** DNA fragmentation and apoptotic population were suppressed in 7-4 derivatives expressing dominant negative *raf*-1. Cell maintenance and treatment were the same as described in Figure 9. (**A**) DNA fragmentation analysis. (**B**) Quantification of apoptotic cells and Ha-*ras* overexpression (1.95- to 2.8-fold) further enhanced this suppression by 1.8- to 2-fold (Figure 7B, lanes 2 and 3, 4 and 5). Moreover, the phosphorylation of $p34^{cdc2}$ was not affected by Ha-*ras* overexpression under serum-deprived conditions (unpublished data). The decrease in $p34^{cdc2}$ at the translational level seems to play an important role in disruption of the cell cycle.

### Dominant-negative *ras* and dominant negative *raf*-1 blocked Ha-*ras* overexpression-induced apoptosis at different signal transduction stages

To ensure that Ha-*ras* indeed triggers the apoptosis of 7-4 cells, the dominant negative *ras* [in plasmid pZIPneoAsn17 with a mutation at amino acid 17 (serine to asparagin)] was transfected into 7-4 cells to block Ras activity (Feig and Cooper, 1988). Three stable transfectants Ras 1, Ras 2 and Ras 3 with higher levels of dominant negative *ras* expression show lower AP-1 activity (Figure 8A), lower efficiency of colony formation (Figure 8B) and slower cell growth rate (Figure 8C) compared with the parental 7-4 cells, indicating that dominant-negative *ras* effectively blocked Ras activity. AP-1 consists of the members of the jun and the fos families, which can be activated by RAS; therefore, its activity can be used as the indicator of Ras activity. Moreover, the degree of DNA fragmentation and the population of apoptotic cells were all evidently decreased in these dominant-negative *ras* cell lines (Figure 9A and B), indicating that *ras* indeed plays a major role in 7-4 cell apoptosis.

Raf-1 is an effector of activated Ras and its downstream transducers are MAPK, Erk and Elk (Kauffmann-Zeh et al, 1997). To unveil whether *ras*-triggered apoptosis is through the *raf*-1 signalling pathway, the dominant negative *raf*-1 gene (in plasmid pRafC4B, with a deletion at 3′ end kinase domain) was transfected into 7-4 cells (Bruder et al, 1992). Three cell lines Raf E, Raf F and Raf M stably expressing the *raf*-1 gene were established (data not shown). To determine the activity of Raf-1, its downstream substrate Elk activity was measured by the PathDetect Elk-*trans*-reporter

© Cancer Research Campaign 1998

system (Stratagene). This reporting system contains a pFAElk fusion activator plasmid and a pFRLuc reporter plasmid. The fusion activator plasmid expresses the pathway-specific transactivator protein that consists of the DNA-binding domain of the yeast GAL4 and the activation domain of the Elk transcription factor. Because this fusion activator pFAElk is phosphorylated and activated by Raf-1, its activity reflects the in vivo activation of this kinase and the corresponding signal transduction pathway. The Raf-1 activity, efficiency of colony formation and the rate of cell growth of Raf F and Raf M were decreased to various degrees compared with their parental 7–4 cells, indicating that Raf activity was blocked by the dominant negative *raf*-1 (Figure 10A and C). Comparatively, Raf E showed much less efficiency in suppression of Raf activity, colony formation and cell growth. We found the mRNA level in Raf E cells is the lowest compared with the other two cells lines (data not shown). Correspondingly, the degree of DNA fragmentation and population of apoptotic cells in Raf F and Raf M were much lower than that in Raf E and 7-4 cells, indicating that the *raf*-1 signalling pathway is indeed required for 7-4 cell apoptosis (Figure 11A and B).

### DISCUSSION

It is conceivable that activated Ha-*ras* may alter the interactions between regulatory accessory factors such as Ras-GAP and Ras-GEF, thereby leading to deregulation of one or more members of the GTP-binding *ras* superfamily (Bokoch and Der, 1993), such as *ran*/TC4 (Basu et al, 1992). The product of *ran*/TC4 is necessary for the function of RCC1, which is a gene product involved in coordinating the end of the S-phase and chromosome condensation (Ren et al, 1993). It is also known that inappropriate expression of normal or activated *ras* can interfere with normal cell cycle progression (Feramisco et al, 1984; Mulcahy et al, 1985). Denko et al (1994) have shown that selective induction of the activated Ha-*ras* transgene with IPTG is sufficient to disrupt at least one cell cycle control point by stimulating serum-deprived 212 cells to progress from $G_1$ arrest into S-phase. All the data indicate that activated *ras* can facilitate the transition from $G_1$ to S-phase of the cell cycle. This phenomenon is consistent with our observation of 7-4 cells that accumulated in S-phase while activated Ha-*ras* was overexpressed by IPTG.

The disruption of the normal cell cycle that resulted in the death of the cells has been reported (Rubin et al, 1993). Meikrantz et al (1994) classified the death of S-phase-arrested cells in an asynchronous population into multiple forms of apoptosis. The correlation between S-phase arresting and apoptosis may explain that 7-4 cells with a major population of S-phase-arrested cells showed high mortality, significant DNA fragmentation and strong acridine orange staining. Such cell cycle disruption may result from either gene expression at the wrong time or aberrant expression of normal cell cycle kinases, including activation or inactivation (Rubin et al, 1993). Chen and Pan (1994) reported that in *Xenopus* oocytes, Ha-*ras* oncogene overexpression suppressed the activity but not the expression level of $p34^{cdc2}$, and subsequently led the cell to rest in M-phase. In our study, Ha-*ras* overexpression greatly suppressed $p34^{cdc2}$ expression level and led the cells to accumulate in S-phase.

Many factors may be involved in the process of Ha-*ras*-induced apoptosis, but the relationships largely remain unknown (Whyte and Evan, 1995). The possible mechanisms of Ha-*ras* overexpression-induced apoptosis in 7-4 cells are as follows: (a) overexpression of Ha-*ras* oncogene inactivates cell cycle-related kinases (such as the kinase $p34^{cdc2}$) (Girard et al, 1991); (b) Ha-*ras* overexpression shuts off the expression of 'survival' genes, such as *bcl*-2 in the absence of appropriate growth factors under serum depleted condition and guides cells to death; (c) the unknown factor that triggered the apoptotic pathway was specially activated in 7-4 cells (such as IRF-1) (Tanaka et al, 1994).

Overexpression of the *bcl*-2 gene blocked Ha-*ras*-induced apoptosis but had no effect on Ha-*ras* oncogene expression, indicating that its prevention of apoptosis is downstream in the Ha-*ras* signalling pathway (unpublished data). Moreover, Bcl-2 and $p21^{ras}$ molecules can be co-immunoprecipitated in Jurkat cells, and phosphorylation of the Bcl-2 protein is involved in the prevention of cell apoptosis (Chen and Faller, 1996). However, the precise mechanism by which the *bcl*-2 gene prevents Ha-*ras*-induced apoptosis remains to be determined.

Our results from (a) selective overexpression of activated Ha-*ras* oncogene by IPTG; and (b) specific blockage of Ras activity by dominant negative *ras* or dominant negative *raf* clearly demonstrate that overexpression of activated Ha-*ras* indeed induces 7-4 cells apoptosis under serum-deprived conditions. Activated Ha-*ras*-induced apoptosis was also detected in the other transformant 212 cells. But the degree of apoptosis was less severe compared with 7-4 cells, indicating that factors other than Ha-*ras* activation may also be involved. However, our observation that 7-4 cells proceeded with apoptosis without c-*myc* overexpression (unpublished data) is different from Kauffmann-Zeh's report that both Ha-*ras* and c-*myc* are overexpressed in the apoptosis of the fibroblasts (Kauffmann-Zeh et al, 1997). This contrasting result indicates that c-*myc* overexpression is not an inducing factor but an end-point response of Ha-*ras*-induced apoptosis.

The effect of serum starvation on long-term viability of the cells was observed by measuring the percentage of dye excluding cells that were stained with 0.04% trypan blue at a 24-h interval. We found that, 72 h after starvation, the percentage of viable 7-4 cells is 88% in the absence of IPTG and drops to 50% in the presence of IPTG compared with NIH/3T3 cells (100%), indicating that NIH/3T3 and 7-4 cells could stand long-term starvation (unpublished data). Ha-*ras* gene overexpression makes 7-4 cells more vulnerable to serum deprivation and induces them to apoptosis.

The uncertainty whether nutrient deficiency caused by serum starvation may induce cell apoptosis was clarified by growing the cells in the medium supplemented with 10% AC-2 (Valio, Finland; collostrum, a serum substitute). We found that NIH/3T3 and a lung carcinoma cell line H2981 could grow normally in this kind of medium (unpublished data). In contrast, 7-4 cells with Ha-*ras* overexpression maintained in the same conditions underwent apoptosis, indicating that serum depletion causing nutrient deficiency is not a determining factor of apoptosis. It is consistent with Kulkarni's report that serum starvation only caused minor cell apoptosis (Kulkarni and McCulloch, 1994). The amount of certain growth factors such as IGF-1 in either 0.2% serum or 10% AC-2 may be critical to determine cell survival or death. A detailed study is under way.

Dominant negative *raf*-1 blocked the Raf-1-MAPK pathway and prevented apoptosis of 7-4 cells, indicating that Ha-*ras*-induced apoptosis needs Raf-1-MAPK pathway activity, which is consistent with Kauffmann-Zeh's observation that Raf-1-MAPK is an apoptotic pathway. It seems that the Raf-MAPK pathway is a common pathway for both proliferation and apoptosis. We hypothesize that in NIH/3T3 fibroblasts while Ha-*ras* was overexpressed, serum starvation as an exogenous stress will turn on the apoptotic signal through

© Cancer Research Campaign 1998

Ha-*ras* and *raf*-1 signalling pathways. The fate of the cells will then be executed by downstream specific nuclear transactivators, which are formed after Ha-*ras* activation and activated through dimer formation (such as the pairing of the members of *jun* and *for* families) that activate either survival genes or apoptotic genes. Ha-*ras* overexpression activated downstream from the expression of apoptotic-related genes was sustained by a protein synthesis inhibitor cycloheximide study, which delayed 7-4 cell apoptosis.

In conclusion, our study demonstrates that Ha-*ras*$^{val12}$ overexpression without c-*myc* overexpression can trigger cell apoptosis This apoptosis may use the Raf-1-MAPK pathway to synthesize specific nuclear factors and subsequently to turn on apoptotic-related genes. These events were confirmed by the studies of dominant-negative *ras*, *raf*-1, cycloheximide and *bcl*-2 gene overexpression.

The relationship between Ha-*ras*$^{val12}$ oncogene overexpression and cell apoptosis observed in our laboratory will shed light on the understanding of Ha-*ras* oncogene-related apoptosis and aid in unveiling the factors that lead tumour cells with the expression of activated Ha-*ras* oncogene to programmed cell death.

### ACKNOWLEDGEMENTS

The authors thank Dr SL Chen and Dr YP Tsao for providing technical assistance in cyclin A and B analysis. This work was supported by grants from the National Science Council, Taiwan (NSC83-0412-B006-055 and NSC85-2331-B006-014).

### REFERENCES

Arends MJ, McGregor NJ, Brown EJ and Wyllie AH (1993) Susceptibility to apoptosis is differentially regulated by c-*myc* and mutated Ha-*ras* oncogenes and is associated with endonuclease availability. *Br J Cancer* **68**: 1127–1133

Arends MJ, McGregor NJ and Wyllie AH (1994) Apoptosis is inversely related to necrosis and determines net growth in tumors bearing constitutively expressed *myc*, *ras*, and HPV oncogenes. *Am J Pathol* **144**: 1045–1057

Barbacid M (1987) *ras* genes. *Annu Rev Biochem* **56**: 779–827

Basu TN, Gutman DH, Fletcher JA, Glover TW, Collins FS and Downward J (1992) Aberrant regulation of *ras* proteins in malignant tumour cells from type 1 neurofibromatosis patients. *Nature* **356**: 713–715

Bokoch GM and Der C (1993) Emerging concepts in the Ras superfamily of GTP-binding proteins. *FASEB J* **7**: 750–759

Bruder JT, Heidecker G and Rapp UR (1992) Serum-, TPA-, and *ras* induced expression from AP-1/Ets-driven promoters requires *raf*-1 kinase. *Gene Dev* **6**: 545–556

Chen CT and Pan BT (1994) Oncogenic *ras* stimulates a 96-kDa histone H2b kinase activity in activated *Xenopus* egg extracts. *J Biol Chem* **269**: 28034–28043

Chen CY and Faller DV (1996) Phosphorylation of Bcl-2 protein and association with p21Ras in Ras-induced apoptosis. *J Biol Chem* **271**: 2376–2379

Denko NC, Giaccia AJ, Stringer JR and Stambrook PJ (1994) The human Ha-*ras* oncogene induces genomic instability in murine fibroblasts within one cell cycle. *Proc Natl Acad Sci USA* **91**: 5124–5128

Downward J (1992) Regulatory mechanisms for *ras* proteins. *Biotechnology* **14**: 177–184

Ducoeur L, Wyborski DL and Short JM (1993) Control of gene expression in eucaryotic cells using the *lac* repression system. *Strat Mol Biol* **5**: 70–72

Feig LA and Cooper GM (1988) Inhibition of NIH/3T3 cell proliferation by a mutant *ras* protein with preferential affinity for GDP. *Mol Cell Biol* **8**: 3235–3243

Feramisco JR, Gross M, Kamata T, Rosenberg M and Sweet RW (1984) Microinjection of the oncogene form of the human H-*ras* (T-24) protein results in rapid proliferation of quiescent cells. *Cell* **38**: 109–117

Fernandez A, Fosdick LJ, Marin MC, Diaz C, McDonnell TJ, Ananthaswamy HN and McConkey DJ (1995) Differential regulation of endogenous endonuclease activation in isolated murine fibroblast nuclei by *ras* and *bcl*-2. Oncogene **10**: 769–774

Girard F, Strausfeld U, Fernandez A and Lamb NJC (1991) Cyclin A is required for the onset of DNA replication in mammalian fibroblasts. *Cell* **67**: 1169–1179

Gulbins E, Bissonnette R, Mahboubl A, Martin S, Nishioka W, Brunner T, Baier G, Baier-Bitterlich G, Byrd C, Lang F, Kolesnick R, Altman A and Green D (1995) FAS-induced apoptosis is mediated via a ceramide-initiated RAS signaling pathway. *Immunity* **2**: 341–351

Hermann M, Lorenz HM, Voll R, Gruk M, Woith W and Kalden JR (1994) A rapid and simple method for the isolation of apoptotic DNA fragments. *Nucleic Acids Res* **22**: 5506–5507

Ito M (1984) Microassay for studying anticellular effects of human interferons. *J Interferon Res* **4**: 603–608

Jimenez B, Adrends M, Esteve P, Perona R, Sanchez R, Cajal SR, Wyllie A and Lacal JC (1995) Introduction of apoptosis in NIH3T3 cells after serum deprivation by overexpression of *rho*-p21, a GTPase protein of the ras superfamily. *Oncogene* **10**: 811–816

Kauffmann-Zeh A, Rodriguez-Viciana P, Ulrich E, Gilbert C, Coffer D, Downward J and Evan G (1997) Suppression of c-*myc*-induced apoptosis by Ras signalling through DI(3)K and PKB. *Nature* **385**: 544–548

Kulkarni GV and McCulloch (1994) Serum deprivation induces apoptotic cell death in a subset of Balb/c 3T3 fibroblasts. *J Cell Sci* **107**: 1169–1179

Liu HS, Scrable H, Villaret DB, Lieberman MA and Stambrook PJ (1992). Control of Ha-*ras*-mediated mammalian cell transformation by *Escherichia coli* regulatory elements. *Cancer Res* **52**: 983–989

McEvoy L, Schlegel RA, Williamson P and Buono BJ (1988) Merocyanine 540 as flow cytometric probe of membrane lipid organization in leukocytes. *J Leukocyte Biol* **44**: 337–344

Meikrantz W, Gisselbrecht S, Tam SW and Schlegel R (1994) Activation of cyclin A-dependent protein kinases during apoptosis. *Proc Natl Acad Sci USA* **91**: 3754–3758

Mulcahy LS, Smith MR and Stacey DW (1985) Requirement for *ras* proto-oncogene function during serum-stimulated growth of NIH 3T3 cells. *Nature* **313**: 241–243

Ren M, Drivas G, D'Eustachio P and Rush MG (1993) Ran/TC4: a small nuclear GTP-binding protein that regulates DNA synthesis. *J Cell Biol* **120**: 313–323

Rubin LL, Philpott KL and Brooks SF (1993) The cell cycle and cell death. *Curr Biol* **3**: 391–194

Sakai N, Ogiso Y, Fujita H, Watari H, Koike T and Kuzumaki N (1994) Induction of apoptosis by a dominant negative H-*ras* mutant (116Y) in K562 cells. *Exp Cell Res* **215**: 131–136

Sambrook J, Fritsch EF and Maniatis T (1989) *Molecular Cloning: A Laboratory Manual*. Cold Spring Harbor Laboratory Press: Cold Spring Harbor, NY

Tanaka N, Ishihara M, Kitagawa M, Harada H, Kimura T, Matsuyama T, Samphier MS, Aizawa S, Mak TW and Taniguchi T (1994) Cellular commitment to oncogene-induced transformation or apoptosis is dependent on the transcription factor IRF-1. *Cell* **77**: 829–839

Tsujimoto Y (1989) Overexpression of the human BCL-2 gene product results in growth enhanceent of Epstein-Barr virus-immortalized B cells. *Proc Natl Acad Sci USA* **86**: 1958–1962

Wang HG, Millan JA, Cox AD, Der CJ, Rapp UR, Beck T, Zha H and Reed JC (1995) R-Ras promotes apoptosis caused by growth factor deprivation via a Bcl-2 suppressible mechanism. *J Cell Biol* **129**: 1103–1114

Wang WB, Bikel I, Marsilid E, Newsome D and Livingston DM (1994) Trans-expression of RNA polymerase II promoters by the simian virus 40 small t antigen. *J Virol* **68**: 6180–6187

Whyte M and Evan G (1995) The last cut is the deepest. *Nature* **376**: 17–18

© Cancer Research Campaign 1998