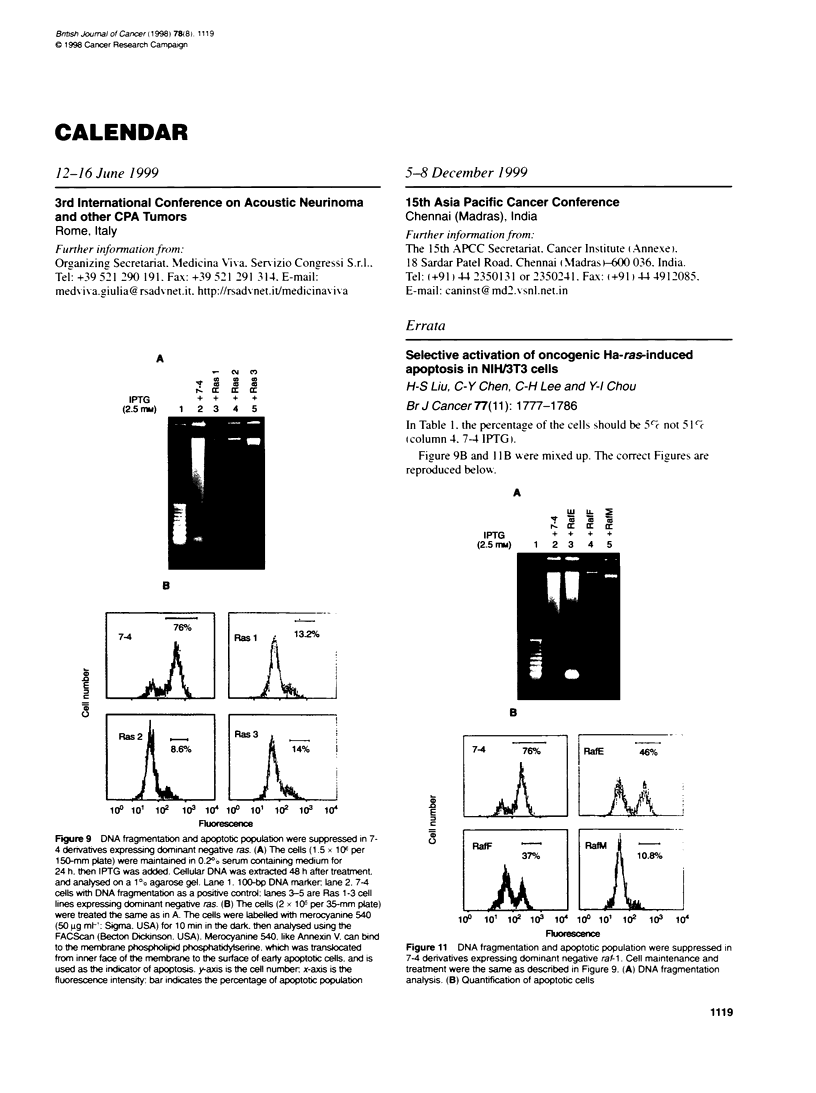# Selective activation of oncogenic Ha-ras-induced apoptosis in NIH/3T3 cells

**Published:** 1998-10

**Authors:** 

## Abstract

**Images:**


### *Errata*

### Selective activation of oncogenic Ha-*ras*-induced apoptosis in NIH/3T3 cells

*H-S Liu, C-Y Chen, C-H Lee and Y-I Chou*

*Br J Cancer* **77**(11): 1777–1786

In Table 1, the percentage of the cells should be 5% not 51% (column 4, 7-4 IPTG).

Figure 9B and 11B were mixed up. The correct Figures are reproduced below.

**Figure 9** DNA fragmentation and apoptotic population were suppressed in 7-4 derivatives expressing dominant negative *ras*. (**A**) The cells ($1.5 \times 10^6$ per 150-mm plate) were maintained in 0.2% serum containing medium for 24 h, then IPTG was added. Cellular DNA was extracted 48 h after treatment, and analysed on a 1% agarose gel. Lane 1, 100-bp DNA marker; lane 2, 7-4 cells with DNA fragmentation as a positive control; lanes 3–5 are Ras 1-3 cell lines expressing dominant negative *ras*. (**B**) The cells ($2 \times 10^5$ per 35-mm plate) were treated the same as in A. The cells were labelled with merocyanine 540 (50 μg $ml^{-1}$; Sigma, USA) for 10 min in the dark, then analysed using the FACScan (Becton Dickinson, USA). Merocyanine 540, like Annexin V, can bind to the membrane phospholipid phosphatidylserine, which was translocated from inner face of the membrane to the surface of early apoptotic cells, and is used as the indicator of apoptosis. *y*-axis is the cell number; *x*-axis is the fluorescence intensity; bar indicates the percentage of apoptotic population

**Figure 11** DNA fragmentation and apoptotic population were suppressed in 7-4 derivatives expressing dominant negative *raf*-1. Cell maintenance and treatment were the same as described in Figure 9. (**A**) DNA fragmentation analysis. (**B**) Quantification of apoptotic cells